# Incomplete Subacute Transverse Myelitis Following Vaccination With Pfizer-BioNTech COVID-19 mRNA Vaccine: A Case Report

**DOI:** 10.7759/cureus.20460

**Published:** 2021-12-16

**Authors:** Jarrah Alabkal, Alexander D Rebchuk, Daniel Lyndon, Nikkie Randhawa

**Affiliations:** 1 Division of Neurology, Faculty of Medicine, University of British Columbia, Vancouver, CAN; 2 Division of Neurosurgery, Faculty of Medicine, University of British Columbia, Vancouver, CAN; 3 Radiology, Faculty of Medicine, University of British Columbia, Vancouver, CAN; 4 Division of Neurology, Vancouver General Hospital, Vancouver, CAN

**Keywords:** pfizer-biontech covid-19 vaccine, bnt162b2, vaccine, mrna vaccine, tozinameran, transverse myelitis, covid-19

## Abstract

In response to the coronavirus disease 2019 (COVID-19) pandemic, rapid development, clinical testing, and regulatory approval of vaccines occurred. The tozinameran COVID-19 vaccine is the first mRNA vaccine approved for use in humans. Transverse myelitis is a rare inflammatory disorder of the spinal cord that is associated with traditional vaccinations. There are rare case reports describing an association between mRNA vaccines and transverse myelitis. Herein, we describe a case of transverse myelitis following mRNA vaccination. A healthy 26-year-old woman developed saddle anesthesia, numbness, and allodynia in the S1-S4 distribution within three days of receiving the first dose of tozinameran COVID-19 vaccine. She had decreased sensation to pinprick, temperature, and light touch in S1-S4 distribution and a positive Rhomberg test. An MRI brain and spine demonstrated a short segment T2 hyperintense and diffusely enhancing lesion at T5. Cerebrospinal fluid studies demonstrated pleocytosis and elevated IgG index. A five-day course of IV methylprednisolone resulted in minimal improvements in her symptoms. Stage III clinical trials may be underpowered to detect more rare adverse effects such as transverse myelitis. Therefore, it is imperative to have ongoing surveillance and reporting of adverse events associated with COVID-19 vaccines to ensure transparency with regard to potential risks to patients obtaining the vaccine and algorithms in place for detection and urgent treatment if required. Nonetheless, the safety and efficacy of vaccination against COVID-19 are well established and greatly outweigh any potential risks associated with the vaccine. Given the individual, societal, and global health benefits of vaccination we strongly advocate for ongoing vaccinations against COVID-19.

## Introduction

Severe acute respiratory syndrome coronavirus 2 (SARS-CoV-2), the causal agent of coronavirus disease 2019 (COVID-19), has infected over 250 million individuals and resulted in millions of deaths globally since it was declared a pandemic in March 2020 by the World Health Organization [1]. In the subsequent months, urgent development and clinical trials of potential vaccines occurred. The tozinameran (Pfizer-BioNTech) vaccine was the first COVID-19 vaccine approved for use by regulatory agencies in the United Kingdom, United States, and Canada [2-4]. Furthermore, it was the first messenger RNA (mRNA) vaccine to be approved for use in humans outside of a clinical trial. Although mRNA vaccines have demonstrated acceptable safety profiles in animal models [5], their long-term safety profile in humans is unknown. Transverse myelitis is a rare inflammatory disorder of the spinal cord. Case reports have established an association between transverse myelitis and traditional vaccinations [6]. There have been early case reports suggesting an association between mRNA vaccines and transverse myelitis [7-8]. Herein, we present a case of post-inoculation transverse myelitis following administration of the Pfizer-BioNTech COVID-19 mRNA vaccine.

## Case presentation

A 26-year-old right-handed woman presented to hospital with progressive saddle anesthesia and bilateral paresthesias, numbness, and intermittent allodynia ascending the plantar aspects of her feet up the posterior legs, extending to the perineum within three days of receiving the first dose of tozinameran vaccine, with further progression over the subsequent eight days with complaints of lack of sensation with defecation, urination, wiping, and sexual intercourse. She has no history of rash, oral or genital ulcers, arthralgia, Raynaud phenomenon, photosensitivity, or other features of systemic or neurologic autoimmune disease. She denied any other neurological complaints, infectious symptoms, history for recent travel, hiking or camping, and sexually transmitted infections. Her medical history was significant for pancreatitis in her adolescence and recurrent urinary tract infections, without urinary retention symptoms, following an unremarkable spontaneous vaginal delivery. She had been treated successfully with antibiotics for an Escherichia coli and Klebsiella urinary tract infection one month prior to her presentation. There was no family history of autoimmune disorders or central nervous system tumors. Her only regular medication was a combination oral contraceptive pill. 

On presentation she was afebrile, had stable vitals, and had a normal abdominal, cardiac, respiratory, genital, and dermatological exam. There was no meningismus and her neck was supple. Her neurological exam was notable for brisk, but symmetric bilaterally, reflexes at biceps, triceps, brachioradialis, patellar and achilles, with a crossed adductor reflex on the right. Sensation to temperature, pinprick, and proprioception was normal in the cervical, thoracic, and lumbar distributions; however, she had symmetrically decreased sensation to pinprick, temperature, and light touch in S1-S4 distribution including the soles of the feet but with posterior leg sparing on objective testing. Her right S1-S2 distribution was slightly more affected than the left. She had blunted sensation with a digital rectal exam, but normal anal sphincter tone, and a positive Rhomberg test. Otherwise, her mental status, cranial nerves, motor, sensory, coordination, and gait were unremarkable. On post-admission day four she complained of right foot weakness and was found to have right extensor hallucis longus strength of 4+/5. This resolved the following day. 

Cerebrospinal fluid (CSF) studies demonstrated pleocytosis and elevated immunoglobulin G (IgG) index (Table 1). CSF bacterial and fungal cultures were negative. Her C-reactive protein (CRP) was elevated at 6.3 mg/L (normal <3.1 mg/L). Otherwise, a complete blood count, extended electrolytes, coagulation, cobalamin, beta-hCG, thyroid panel, rheumatologic panel [antinuclear antibody (ANA), C3, C4, rheumatoid factor], and protein electrophoresis were within normal limits. Testing for Borrelia burgdorferi, syphilis, human immunodeficiency virus (HIV) were negative; however, she was positive for a reactive IgG antibody to varicella-zoster virus. CSF bacterial and fungal cultures, acid-fast bacilli, cryptococcal antigen, HSV, VZV, enterovirus, anti-AQP4-Ab, and anti-MOG-Ab were negative. 

**Table 1:** Cerebrospinal fluid studies performed on presentation to community hospital (post-vaccination day 8) and repeated while at tertiary care academic hospital (post-vaccination day 13). Bolded results indicate abnormal values. n.p., not performed; WBC, white blood cell; RBC, red blood cell; IgG, immunoglobulin

Study	Post-vaccination day 8	Post-vaccination day 13	Normal range
Glucose	3.3	3.0	2.3-4.1 mmol/L
WBC	9	19	0-5 x10^6^/L
Protein	0.32	0.34	0.15-0.45 g/L
RBC	<500	<500	0-5 x10^6^/L
Neutrophils	0	n.p.	<1%
Lymphocytes	100	98	63%-99%
Mononuclear cells	n.p.	100	70%-100%
Monocytes	n.p.	2	3%-37%
IgG Index	n.p.	0.68	0.34-0.66

**Figure 1:**
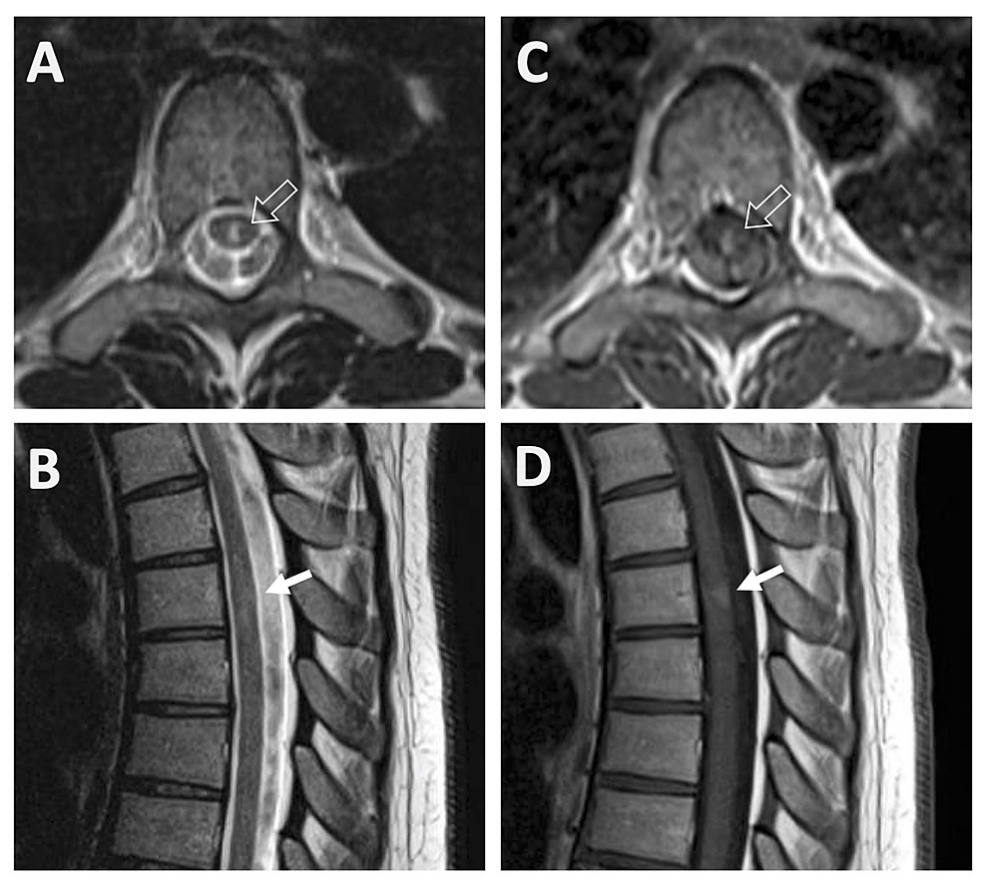
Images (A) and (B): Axial and sagittal T2-weighted sequences showing a centrally located short segment T2 hyperintense cord lesion without associated mass effect. This is less well seen on the sagittal image due to its narrow transverse diameter. Images (C) and (D): Post-gadolinium T1-weighted images showing moderate enhancement of the same lesion.

An MRI of her entire spinal cord demonstrated a short segment T2 hyperintense and diffusely enhancing lesion at T5 (Figure 1), in keeping with transverse myelitis. An MRI head demonstrated three non-specific small patchy T2 hyperintense lesions within the supratentorial deep white matter (right anterior frontal lobe, right frontal operculum, and left internal capsule) which were felt to be nonspecific.

Her clinical picture was suggestive of incomplete subacute transverse myelitis, therefore, she was given a five day course of IV methylprednisolone (1000 mg daily) beginning 10 days after presentation (post vaccination day 18). She had initially been started on pregabalin (25 mg twice daily), prior to imaging and CSF studies, without any response. Her paresthesia slightly improved with steroid treatment particularly in her left foot, however, she remained symptomatic on discharge. This case was reported to the Provincial Medical Health Officer as an adverse event following vaccination. 

## Discussion

Transverse myelitis occurs when an immune-mediated inflammatory process causes neural injury to the spinal cord, leading to varying degrees of weakness, sensory alterations, and autonomic dysfunction [9-10]. There have been case reports of transverse myelitis associated with vaccines for hepatitis B, polio, diphtheria, tetanus, pertussis, influenza, rabies, measles, mumps, rubella, typhoid, Japanese encephalitis, anti-cholera, and H1N1 [6, 11]. However, a causal relationship between acute demyelinating events, including transverse myelitis or acute disseminated encephalomyelitis, and vaccinations remains inconclusive. This is partly related to the rarity of these disorders and the need for larger populations to be studied with adequate statistical power. In addition, incidence rates of these disorders peak during early and middle adulthood when fewer vaccines of any kind are being administered in comparison to young children and older adults [12].

Vaccine development requires several sequential steps taking place over the course of years to be deemed effective and safe to progress through clinical trial phases and ultimately integrated into widespread use. However, COVID-19 vaccine development has accelerated at an unparalleled pace. This is primarily due to factors including, but not limited to, high demand given the associated morbidity and mortality, and economic implications of a prolonged pandemic, as well as immediate available funding and participants through cooperative global efforts toward vaccine development. There are different vaccine platforms of which the mRNA vaccine was the first to be produced for SARS-CoV-2 representing a new approach. Compared to traditional vaccines, mRNA vaccines can be developed more rapidly, using a synthetic manufacturing process, and provide more versatility in pathogen targeting [5].

An mRNA vaccine mimics a viral infection to trigger an in situ host antigen response and thus elicits a potent humoral and cellular response [5]. Vaccines can induce autoimmunity through molecular mimicry between infectious antigens and self-antigens, epitope spreading whereby invading antigens accelerate an ongoing autoimmune process by local activation of antigen-presenting cells and over-processing of antigens, polyclonal activation of B lymphocytes, or bystander activation which enhances cytokine production and further induce the expansion of auto reactive T-cells [6, 13-16]. Additionally, mRNA vaccines may activate several pro-inflammatory cascades [17]. We suspect that up-regulation of these immunological pathways is the basis for transverse myelitis observed following administration of the tozinameran COVID-19 vaccine in the presented case. Interestingly, in our case, short segment focal disease at T5 was observed on MRI. Although long segment involvement is most commonly described in cases of vaccine-associated transverse myelitis, a recent literature review of case reports describing acute transverse myelitis following vaccine administration found variable cord segment involvement, including cases with single-segment involvement [18].

In stage III clinical trial for BNT162b2, of 18,860 patients receiving the vaccine, four (<0.01%) developed serious adverse events related to the vaccine (soft tissue shoulder injury related to vaccine administration, ipsilateral axillary lymphadenopathy, paroxysmal ventricular arrhythmia, and transient leg paresthesia) [12]. In the patient who reported transient leg paresthesia, a diagnosis of transverse myelitis was not made and it is unclear if any specific neuroimaging or CSF analysis was undertaken [12]. In the Oxford-AstraZeneca COVID-19 vaccine (ChAdOx1 nCoV-19 vaccine; AZD1222, a chimpanzee adenovirus-vectored vaccine) efficacy study there was one case of transverse myelitis 14 days after booster vaccination which was deemed possibly related, and diagnosed as an idiopathic short segment, spinal cord demyelination by the independent neurological review committee [19].

The stage III clinical trial for BNT162b2, while demonstrating significant efficacy and only mild "transient reactogenicity" may have been underpowered to detect more rare adverse effects such as transverse myelitis. Furthermore, variable follow-up time after dose one for adverse event analyses was used, which may have missed later-onset neurological adverse events which may become more evident as more safety monitoring data are collected up to two years post-second dose. Similarly, no subgroup analysis was performed based on age, sex, ethnicity, BMI, or pre-existing underlying condition, which may have included a lower proportion of patients who may be at higher risk for post-vaccination autoimmune sequelae. As such, close surveillance of potential adverse neurologic effects is essential to better understand the safety profile of these vaccines, especially with regard to rare adverse events such as transverse myelitis. This is even more important as these COVID-19 vaccines are introduced into the general population and likely integrated into the yearly flu vaccine to manage evolving strains. 

## Conclusions

To our knowledge, this is the first reported case of transverse myelitis temporally associated with the tozinameran (Pfizer-BioNTech) Covid-19 vaccine. Our case suggests that transverse myelitis may be associated with mRNA vaccines, however, further study and monitoring are required before concluding that a definitive association exists. Similar to traditional vaccines, mRNA vaccines might trigger autoimmune demyelination presenting as transverse myelitis. Given the rare occurrence of transverse myelitis and the need for very large populations to study them with significant statistical power, it is difficult to establish a relationship between tozinameran and acute demyelinating events. As such, it is imperative to have ongoing surveillance and reporting of adverse events associated with COVID-19 vaccines to ensure transparency with regard to potential risks to patients obtaining the vaccine and algorithms in place for detection and urgent treatment if required. Nonetheless, it should be emphasized that the safety and efficacy of vaccination against COVID-19 are well established and greatly outweigh any potential risks of neurological adverse events associated with the vaccine. Given the individual, societal, and global health benefits of vaccination we strongly advocate for ongoing vaccinations against COVID-19.
